# Association Between Self‐Reported Psychiatric Diseases and Oral Health Among Older Adults

**DOI:** 10.1111/scd.70180

**Published:** 2026-05-06

**Authors:** Humberto Alexander Baca Juárez, Luísa de Souza Maurique, Pedro Paulo de Almeida Dantas, Larissa Viana de Oliveira, Gabriele Winter Santana, Paulo Roberto Grafitti Colussi, Francisco Wilker Mustafa Gomes Muniz

**Affiliations:** ^1^ Graduate Program in Dentistry Federal University of Pelotas Pelotas Brazil; ^2^ Cyro Martins Psychiatric Association Porto Alegre Rio Grande do Sul Brazil; ^3^ School of Dentistry Federal University of Pelotas Pelotas Brazil; ^4^ Private Clinic, University of Passo Fundo Passo Fundo Rio Grande do Sul Brazil; ^5^ Department of Periodontology School of Dentistry Federal University of Pelotas Pelotas Brazil

**Keywords:** frail elderly, mental health, psychiatric disorder, public health, tooth loss

## Abstract

**Aims:**

This study assessed the association between self‐reported psychiatric diseases and the number of present teeth in older adults.

**Methods:**

It was included 569 older adults selected using a probabilistic per cluster sampling strategy. Structured questionnaire was applied, and an oral health examination was performed. Self‐reported psychiatric diseases were defined as the primary exposure, while the number of present teeth was the outcome. Adjusted analyses were performed using Poisson regression with robust variance (*α* < 0.05).

**Results:**

The prevalence of self‐reported psychiatric disease was 16.5% (*n* = 94; 95% confidence interval [95% CI]: 13.5%–19.6%). In the final adjusted model, lower number of present teeth was associated with self‐reported psychiatric diseases (rate ratio [RR]:0.69; 95% CI:0.51–0.94). Women (RR:0.72; 95% CI:0.61–0.84), those without access to the dentist (RR:0.54; 95% CI:0.45–0.65), and of higher age (RR:0.96; 95% CI:0.94–0.97) also presented lower number of present teeth when compared to their counterparts. Those with medium level of education presented higher number of teeth (RR:1.68; 95% CI: 1.43–1.98).

**Conclusions:**

Older adults with self‐reported psychiatric diseases, females, and those without dental access exhibited higher rates of tooth loss. Public policies should aim to integrate mental and oral health, ensuring equitable access to dental care for these most vulnerable groups.

## 1 Introduction

Population aging is one of the major sociodemographic shifts of the 21^st^ century, increasing the burden of chronic conditions among older adults [1, 2]. Mental health is a key component of healthy aging, and recent global analyses indicate that 15%–30% of individuals aged 60 years or older experience mental disorders, particularly depression and anxiety [3, 4]. Systematic reviews reinforce the high prevalence of these conditions and their impact on overall well‐being [5, 6].

According to the Diagnostic and Statistical Manual of mental disorders‐5 (DSM‐5), a psychiatric disorder is a clinically significant disturbance in an individual's cognition, emotion regulation, or behavior that reflects a dysfunction in the psychological, biological, or developmental processes underlying mental functioning [7]. Mental disorders in older adults are associated with poorer general health and reduced engagement in preventive behaviors, including oral hygiene [8]. Tooth loss remains highly prevalent worldwide and affects mastication, aesthetics, social interaction, and quality of life [9]. These factors make oral health an important component of functional and psychological well‐being in aging populations.

Evidence shows that individuals with psychiatric disorders tend to present worse oral health outcomes, including higher prevalence of periodontal disease, dental caries, and tooth loss. This association may be influenced both by behavioral factors and by adverse effects of psychotropic medications, such as xerostomia, which increases susceptibility to oral diseases [10]. Recent reviews highlight that older adults with mental disorders face multiple barriers to maintaining adequate oral hygiene [11].

However, data on the relationship between psychiatric conditions and oral health among older adults in Brazil are still scarce [12]. Understanding this association is essential to guide public health strategies directed at vulnerable aging populations. Therefore, this study aimed to evaluate the association between self‐reported psychiatric diseases and the number of present teeth among older adults from two cities in Southern Brazil. We hypothesized that individuals with psychiatric conditions would present fewer remaining teeth.

## 2 Methods

### 2.1 Study Design, Location, and Ethical Aspects

This is a cross‐sectional study that interviewed and examined older adults aged 60 years or older in two cities of Brazil, Veranópolis and Cruz Alta. In Veranópolis, individuals aged at least 60 years in both urban and rural areas were randomly selected. This city is located in the northeast of the state, 160 km from the capital, Porto Alegre. Veranópolis has 22 810 inhabitants, of which 3554 are over 60 years old, 42.91% are male and 57.09% are female [13]. The Municipal Human Development Index (MHDI) in 2010 was 0.75 [14]. In the city of Cruz Alta, older adults aged 65–74 years living in the urban area were selected. The city is located in the northern region of the state, 350 km from the capital. Cruz Alta has 62 821 inhabitants, 3,730 are in this age group, 42% men, and 58% women [13]. The MHDI, in 2010, was 0.75 [14].

For both cities, different study protocols were approved by the local Ethics Committee under the protocols #1,531,862 and #2,990,088. All participants read and signed the informed consent form before participating in the study. Measures were taken to ensure the privacy and confidentiality of participants, such as anonymizing all collected data and storing it securely to prevent unauthorized access, as well as identifying each of them with an ID on the collection forms. The present study followed the STROBE checklist [15]. Data collection occurred from July to August 2016 in Cruz Alta and from December 2018 to January 2019 in Veranópolis.

### 2.2 Sampling Strategy

To be representative of the two cities, different sampling strategies were performed. Further explanations, regarding the sampling strategy, and may be found elsewhere [16, 17]. Participants were selected using a probabilistic per cluster sampling strategy. The areas from both cities were randomly divided and chosen using specific software (www.random.org). In the city of Veranópolis, older adults aged 60 or over from urban and rural areas were included. Eighty‐seven percent (87%) of them lived in urban areas. For this reason, the rural area was also included. Eighty‐two areas (20% of the total areas) and three rural areas (out of a total of 21 rural communities) were randomly selected.

In the city of Cruz Alta, only individuals aged 65–74 years were included, as this age group represents a significant proportion of the elderly population and aligns with the focus of this study. More than 95% of residents lived in an urban area, thus, only individuals living in that area were included. To ensure representativity, seventeen urban areas were randomly selected, respecting the proportion of elderly residents in each area based on official demographic data.

For both cities, using the city map, each selected area was divided into blocks. The blocks were also randomly selected. Moreover, the corners of each block were numbered to randomly select the starting point of the visit to the householdings. Afterwards, the interviews were performed in a clockwise manner. In each selected area, households were visited until a sufficient number of individuals were included. When necessary, a new block was randomly selected until a sufficient number of individuals were included.

### 2.3 Inclusion and Exclusion Criteria

In both cities, the inclusion criteria were noninstitutionalized older adults, individuals had physical, medical, and mental conditions that allowed them to answer all the questions asked by the researchers and receive the exams. In each household, if more than one older adult met the inclusion criteria, all of them were invited to participate. In the buildings, only one apartment was included in the present study. Households were excluded only after two attempts of data collection. In addition, visitors, long‐term residences for the older adults, commercial establishments, and uninhabited houses were excluded.

### 2.4 Clinical Examination and Interview

The same structured questionnaire was applied in both cities, which included sociodemographic, behavioral, medical, and dental history variables. These questions were obtained from a block of questions by the PCA‐Tool‐ Brazil [18]. The PCA‐Tool‐Brazil questionnaire has been previously validated for use in similar populations, ensuring reliability and accuracy in data collection [19]. For each city, two teams of researchers, which included an interviewer, a note taker, and an oral health, were sent. All of them were supervised by the same coordinating researcher.

The oral health variable was composed by the number of teeth present. Clinical oral examinations were performed, after the interview, with a wooden spatula and natural illumination. All teeth were counted, except for third molars. Teeth that could somehow be restored were counted, while roots were considered missing.

Before starting the study, all researchers were trained and, when applicable, and calibrated. Training consisted of lectures on the subject of the study, discussion of all items in the questionnaire and explanation of oral health examination. Training was performed for the application of the questionnaire and the oral health examination with older adults undergoing treatment at the clinics of the School of Dentistry of the University of University of Passo Fundo. Calibration was performed with these patients, but none of them were included in the study. Approximately 5% of the total sample were examined twice with at least two days apart. When considering all examiners, the kappa (κ) coefficient for inter‐ and intra‐examiner reproducibility showed a high Kappa index for the number of teeth present in the mouth (kappa indexes were 0.85 (Cruz Alta, intra‐examiner), 0.80 (Cruz Alta, inter‐ examiner), and 0.89 (Veranópolis both inter‐ and intra‐examiner).

### 2.5 Outcome Definition

Number of teeth present was defined as the main outcome and determined through clinical examination, counting all teeth present. Oral clinical examinations were performed with a wooden spatula after the interview, and all teeth, except the third molars, were counted. Natural teeth that had been treated or could be treated/restored in any way were considered present, while roots (or any tooth that the evaluator considered lost), dental implants, and/or dentures were considered absent.

### 2.6 Primary Exposure

Self‐reported psychiatric diseases were defined as the primary exposure. The mental health status was measured by the questionnaire through the question: “Do you have any health problems, such as a psychiatric illness?” The response rates were “yes” or “no.” However, no distinction for what psychiatric diseases was collected. Based on that, the sample was dichotomized.

### 2.7 Other Independent Variables

Other independent variables included: age (measured in completed years at the time of the interview); sex (male or female, based on self‐report); ethnicity/skin color (self‐reported, categorized as white or non‐white, with the non‐white group including individuals identifying as black, yellow, brown, or Indigenous); level of education (low, for those with incomplete primary education or illiterate, and medium/high, for those with at least incomplete or complete secondary education); marital status (married or not, with the latter group including widowed, single, or divorced individuals); retirement (yes or no, based on self‐report); exposure to smoking (categorized as current smokers, ex‐smokers, and nonsmokers); exposure to alcohol (assessed through self‐report and categorized as yes for current alcohol use or no for no alcohol use at the time of the study); and access to the dentist in the last 12 months (yes or no, based on self‐report).

### 2.8 Statistical Analysis

Univariate analysis, for association between all variables with self‐report psychiatric diseases, was performed using the Chi‐square (*χ* ^2^) test (for categorical variables) or Mann–Whitney test (for continuous variables). A nonparametric test was used, as an asymmetric distribution was identified for all continuous variables (including the outcome) collected in the present study, as assessed by the Shapiro–Wilk test.

Bi‐ and multivariable Poisson regression with robust variance were performed to estimate the rate ratio (RR) and its 95% confidence interval (95% CI) for the tested associations. Only the variables that presented a *p*‐value < 0.20 in the bivariable analysis were included as co‐variables in the initial multivariate model. The self‐report psychiatric diseases were included in the final adjusted model regardless of the *p*‐value. To assess the robustness of the findings, sensitivity analyses were performed considering the participants’ city of origin and sex as a subgroup. All analyses were performed in the SPSS software (version 29.0, for Mac, IBM Corp., Armonk, NY, USA), using an *α* < 0.05 to determine the statistical significance of all analyses. Furthermore, analyses of effect modifications were used to define the final multivariate model. Analysis of multicollinearity between independent variables was performed and none was observed.

## 3 Results

When considering the two cities, 569 older adults were included in the present study. Self‐reported psychiatric diseases were reported by 94 individuals, obtaining a prevalence of 16.5% (95% CI:13.5%–19.6%). In the frequency distribution analysis (Table 1), women (*p* < 0.001) and those with low level of education (*p* = 0.032) showed significant associations with the outcome. Regarding the oral health, it was possible to observe a significantly lower number of teeth present in those individuals who reported the presence of psychiatric diseases (4.92 ± 7.35) compared to those without this condition (8.37 ± 8.82).

**TABLE 1:** Distribution of frequency and univariate analysis for the association between self‐reported psychiatric diseases and independent variables.

Variables		No psychiatric diseases (*n* = 475; 83.5%)	Psychiatric diseases (*n* = 94; 16.5%)	*p*‐value
Sex	Male—*n* (%)	167 (35.2)	16 (17.0)	<0.001*
	Female—*n* (%)	308 (64.8)	78 (83.0)	
Age	Mean ± SD	70.26±5.99	70.83±7.08	0.464†
Skin color	White—*n* (%)	367 (77.3)	81 (86.2)	0.054*
	Non‐white—*n* (%)	108 (22.7)	13 (13.8)	
Level of education	Low—*n* (%)	338 (71.2)	77 (81.9)	0.032*
	Medium/high—*n* (%)	137 (28.8)	17 (18.1)	
Marital status	Married—*n* (%)	271 (57.1)	44 (46.8)	0.068*
	Not married—*n* (%)	204 (42.9)	50 (53.2)	
Retirement	Yes—*n* (%)	387 (81.5)	81 (86.2)	0.276*
	No—*n* (%)	88 (18.5)	13 (13.8)	
Smoking exposure	Smokers—*n* (%)	46 (9.7)	9 (9.6)	0.153*
	Former smokers—*n* (%)	136 (28.6)	18 (19.1)	
	Never smokers—*n* (%)	293 (61.7)	67 (71.3)	
Access to the dentist in the last 12 months	Yes—*n* (%)	224 (47.2)	42 (44.7)	0.660*
	No—*n* (%)	251 (52.8)	52 (55.3)	
Alcohol exposure	Yes—*n* (%)	229 (48.2)	37 (39.4)	0.116*
	No—*n* (%)	246 (51.8)	57 (60.6)	
Number of present teeth	Mean ± SD	8.37±8.82	4.92 ± 7.35	<0.001†

*Note*: Legend: *Chi‐square (χ^2^) test; †Mann–Whitney test. Values in bold indicate statistical significance.

Table 2 shows the findings of the bi‐ and multivariable analyses. In the bivariable analysis, older adults with self‐reported psychiatric diseases had 41% fewer teeth present compared with those without such conditions (RR: 0.59; 95% CI: 0.43–0.80). Similar patterns of disadvantage were observed among women, older individuals, those not married, and those who had not visited a dentist in the past 12 months. Smoking exposure did not show statistically significant associations.

**TABLE 2:** Bi‐ and multivariable analyses, using Poisson regression, for the association between number of present teeth with self‐reported psychiatric diseases and other independent variables.

Variables		Bivariable analysis		Multivariable analysis	
		RR (95%CI)	*p*‐value	RR (95%CI)	*p*‐value
Sex	Male	1		1	
	Female	0.65 (0.54–0.77)	<0.001	0.72 (0.61–0.84)	<0.001
Age		0.94 (0.93–0.96)	<0.001	0.96 (0.94–0.97)	<0.001
Skin color	White	1			
	Non‐white	1.00 (0.81–1.24)	1.000	—	—
Level of education	Low	1		1	
	Medium/high	2.22 (1.87–2.63)	<0.001	1.68 (1.43–1.98)	<0.001
Marital status	Married	1			
	Not married	0.66 (0.54–0.80)	<0.001	—	—
Retirement	No	1			
	Yes	1.02 (0.80–1.30)	0.859	—	—
Smoking exposure	Smokers	1			
	Former smokers	1.09 (0.77–1.53)	0.629		
	Never smokers	1.04 (0.76–1.44)	0.791	—	—
Alcohol exposure	Yes	1			
	No	0.74 (0.61–0.89)	0.001	—	—
Access to the dentist in the last 12 months	Yes	1	<0.001	1	<0.001
	No	0.44 (0.36–0.53)		0.54 (0.45—0.65)	
Self‐reported psychiatric diseases	No	1	<0.001	1	0.018
	Yes	0.59 (0.43–0.80)		0.69 (0.51–0.94)	

*Note*: Legend: Initial multivariate model included the following variables: Sex, age, level of education, marital status, alcohol exposure, access to the dentist, and self‐reported psychiatric diseases. Values in bold indicate statistical significance.

In the adjusted model, psychiatric diseases remained significantly associated with tooth loss. Participants reporting these conditions presented 31% fewer teeth than those without psychiatric diseases (RR: 0.69; 95% CI: 0.51–0.94). Although alcohol exposure and marital status did not persist in the final model, all other variables maintained trends consistent with the crude analyses.

In the sensitivity analyses (Table 3), the overall pattern of associations remained consistent with the main findings. Age, educational level, and recent dental attendance showed similar directions and magnitudes across both cities and sexes, reinforcing the robustness of these associations. The effect of sex was significant only in Cruz Alta, mirroring the trend observed in the global analysis. Regarding psychiatric conditions, the association persisted specifically among participants from Veranópolis and among women, suggesting that the impact of psychiatric diseases on tooth loss may be more pronounced in these subgroups.

**TABLE 3:** Sensitivity analyses (adjusted analyses), using Poisson regression, and for the association between number of present teeth with self‐reported psychiatric diseases and other independent variables considering the city of origin (Cruz Alta or Veranópolis) and sex (male or female).

Variables		Cruz Alta		Veranópolis		Male		Female	
		RR (95% CI)	*p*‐value	RR (95% CI)	*p*‐value	RR (95% CI)	*p*‐value	RR (95% CI)	*p*‐value
Sex	Male Female	Ref. 0.68 (0.56–0.83)	<0.001	Ref. 0.78 (0.61–1.01)	0.053	—	—	—	—
Age		0.96 (0.93–0.99)	0.012	0.96 (0.94–0.97)	<0.001	0.97 (0.95–0.99)	0.001	0.95 (0.93–0.97)	<0.001
Level of education	Low Medium/High	Ref. 1.63 (1.33–2.01)	<0.001	Ref. 1.79 (1.38–2.33)	<0.001	Ref. 1.50 (1.20–1.87)	<0.001	Ref. 1.83 (1.45–2.30)	<0.001
Access to the dentist in the last 12 months	Yes No	Ref. 0.61 (0.48–0.76)	<0.001	Ref. 0.46 (0.34–0.62)	<0.001	Ref. 0.60 (0.46–0.77)	<0.001	Ref. 0.51 (0.40–0.66)	<0.001
Self‐reported psychiatric diseases	No Yes	Ref. 0.86 (0.58–1.27)	0.444	Ref. 0.59 (0.38–0.92)	0.020	Ref. 0.88 (0.52–1.51)	0.641	Ref. 0.64 (0.45–0.91)	0.013

Abbreviations: Legend: RR, Rate ratio; 95%CI, 95% confidence interval.

## 4 Discussion

This study aimed to assess the association between tooth loss and self‐reported psychiatric diseases among older adults. A moderate prevalence of psychiatric conditions was observed among older adults, particularly in those with a lower number of present teeth, female individuals, and those who self‐reported being white. The global prevalence of depressive symptoms in older adults is estimated at 35.1% (95% CI: 30.2–40.0) [5], with higher rates in low‐ and middle‐income countries, such as Brazil, where the prevalence reaches 41.3% (95% CI: 34.0–49.0). These findings highlight the importance of studies investigating mental health and associated factors among older adults to inform targeted interventions.

The literature consistently reports a high prevalence of oral health‐related quality of life impacts among individuals with psychiatric disorders, including schizophrenia and depression, and often due to caries experience [20]. Poorer oral health status has also been documented in individuals with mental health disorders. For instance, those treated with fluoxetine exhibited 48% lower odds of bleeding on probing compared to individuals with untreated clinical depression [21]. Furthermore, older adults generally present a higher prevalence and extent of periodontitis compared to the general population [22], with dental caries and periodontal diseases recognized as leading causes of tooth loss. Thus, the lower number of teeth observed in older adults with psychiatric conditions in the present study may be attributed to the greater burden of oral diseases in these individuals.

Pharmacological treatment for psychiatric disorders may also contribute to poor oral health outcomes. Many medications used to treat anxiety, depression, bipolar disorder, and schizophrenia, particularly tricyclic antidepressants, are known to induce dry mouth as a side effect [23]. Additionally, polypharmacy and depression, which are highly prevalent among older adults, are associated with dry mouth [24], increasing susceptibility to dental caries and fungal infections [25]. The challenges in achieving optimal periodontal treatment outcomes in older individuals, as compared to younger populations [26], may further exacerbate the risk of tooth loss in those with psychiatric conditions.

Additionally, diabetes is a relevant factor influencing oral health. Individuals with diabetes have a 63% higher risk of tooth loss compared to those without the condition [27]. The burden of diabetes is also age‐related, with a prevalence of 25% among older adults in the United States [28] and 20% in Brazil [29]. Given that polypharmacy among older adults significantly impacts mortality rates [30], studies examining the interrelations between systemic conditions, psychiatric disorders, and oral health are highly relevant.

The present study also found a higher self‐reported occurrence of psychiatric conditions among female older adults, consistent with prior research indicating that the prevalence of major depression is twice as high among females [31]. This aligns with extensive literature indicating poorer mental health status among women [32]. One possible explanation is that women may be more inclined to express emotions and seek help for psychiatric conditions, whereas men may suppress these feelings. These gender differences in the expression and reporting of mental health conditions may explain the higher prevalence observed among female older adults in the present study. In addition, the gender differences observed in our study may reflect not only biological or behavioral patterns, but also well‐documented cultural differences in how men and women report symptoms and engage with health services. Evidence shows that women tend to recognize and report psychological symptoms more readily than men, whereas men are more likely to underreport mental health problems due to social norms and gender‐role expectations [33, 34]. These factors may have influenced the self‐reporting of psychiatric.

The study's limitations should be acknowledged. The reliance on self‐reported psychiatric conditions introduces the potential for recall bias and perception bias, particularly because the question captured only current diagnoses and did not differentiate between specific psychiatric disorders or past conditions, which could underestimate prevalence and dilute associations. Additionally, the absence of validated mental health assessment tools and the lack of differentiation between specific psychiatric conditions limit the accuracy of the findings. Selection bias may also be present, as individuals with more severe psychiatric disorders might have been underrepresented. The cross‐sectional design further restricts causal inferences, as it does not establish temporality between psychiatric conditions and oral health outcomes. Moreover, the inclusion of only two cities must be considered, as the present study does not aim to be representative for the whole country. Further studies should incorporate validated diagnostic instruments, longitudinal designs, and more comprehensive assessments of mental health status and lifestyle factors to strengthen the evidence base.

Differences between the two participating cities, such as age distribution and the inclusion of urban versus rural areas in one city only, could affect comparability. However, this limitation was mitigated through age‐adjusted analyses and by conducting a sensitivity analysis stratified by city of origin, which yielded consistent results. Also, it is important to note that the main exposure variable (“self‐reported psychiatric diseases”) encompassed a broad range of conditions, as no information regarding the specific type of psychiatric disorder was collected. This lack of diagnostic specification introduces heterogeneity within the exposure category and should be considered when interpreting the findings.

Another limitation is that the question used to assess psychiatric illnesses only asked whether participants had any psychiatric illness at the time of data collection. Thus, the instrument did not capture previous or unknown psychiatric conditions. By restricting the assessment to current disorders, there is a risk of underestimating the true prevalence of psychiatric conditions and of failing to detect relevant associations with oral health outcomes. Although some p‐values are borderline, a posteriori power calculation for our primary outcome demonstrated sufficient power (98.01%), reinforcing the robustness of the main findings. The other results, however, being secondary analyses, should be interpreted with greater caution, as they may reflect sampling limitations.

Finally, as institutionalized or severely ill older adults were not included in the sample, the prevalence of psychiatric conditions may have been underestimated, and the associations observed may not fully represent more vulnerable subgroups. Further studies, specifically designed for these populations, should be performed.

Nevertheless, the study presents important strengths. The randomization and representativeness of the sample enhance the external validity of the findings. Additionally, the use of calibrated oral health examiners ensures reliability in assessing the number of present teeth. These methodological strengths support the robustness of the observed associations.

In conclusion, a high prevalence of self‐reported psychiatric disorders was identified among older adults. This condition was associated with female gender, white skin color, and a lower number of present teeth. These results highlight a critical and often underrecognized public health issue, demonstrating the urgent need for targeted mental health screening, integrated oral and mental health care, and interdisciplinary strategies to improve overall well‐being in this vulnerable population.

## Author Contributions

Francisco Wilker Mustafa Gomes Muniz: Conceptualization, Formal analysis, Methodology, Supervision, Writing – review and editing. Gabriele Winter Santana: Methodology, Validation, Writing – review and editing. Humberto Alexander Baca Juárez: Investigation, Methodology, Visualization, Writing – original draft. Larissa Viana de Oliveira: Methodology, Validation, Writing – review and editing. Luísa de Souza Maurique: Data curation, Validation, Writing – original draft. Paulo Roberto Grafitti Colussi: Conceptualization, Data curation, Funding acquisition, Investigation, Methodology, Writing – review and editing. Pedro Paulo de Almeida Dantas: Methodology, Validation, Writing – original draft.

## Funding

This study was financed in part by the CAPES—Coordenação de Aperfeiçoamento de Pessoal de Nível Superior—Brasil—Finance Code 001. All other funding was self‐supported by the authors.

## Conflicts of Interest

The authors declare no conflicts of interest.

## Data Availability

Data will be available upon request.

## References

[1] World Health Organization [Homepage on the Internet], Ageing and Health. World Health Organization. (2024), [Cited 2025 Nov 22]. https://www.who.int/news‐room/fact‐sheets/detail/ageing‐and‐health.

[2] Y. Gao , W. Zhou , Q. Wang , Q. Zhai , and L. Zhou , “Global, Regional, and National Burden of Mental Disorders and Substance Use Disorders in Older Adults From 1990 to 2021: A Systematic Analysis and Future Trend Prediction Study.” Frontiers in Psychiatry 16 (2025): 1638646. 41069953 10.3389/fpsyt.2025.1638646 PMC12504471

[3] Y. Chen , X. Dong , W. Xu , Y. Long , and K. Liu , “Global Trends and Health Inequalities in Mental Disorders Among Older Adults: A Comprehensive Analysis Using Global Burden of Disease 2021 Data,” Frontiers Public Health 13 (2025): 1644610. 10.3389/fpubh.2025.1644610 PMC12419478 40933400

[4] A. Jalali , A. Ziapour , Z. Karimi , et al., “Global Prevalence of Depression, Anxiety, and Stress in the Elderly Population: A Systematic Review and Meta‐Analysis,” BioMed Central Geriatrics 24 (2024): 809. 39367305 10.1186/s12877-024-05311-8 PMC11451041

[5] H. Cai , Y. Jin , R. Liu , Q. Zhang , Z. Su , and G. S. Ungvari , “Global Prevalence of Depression in Older Adults: A Systematic Review and Meta‐Analysis of Epidemiological Surveys,” Asian Journal of Psychiatry 80 (2023): 103417. 36587492 10.1016/j.ajp.2022.103417

[6] A. Shafiee , I. Mohammadi , S. Rajai , K. Jafarabady , and A. Abdollahi , “Global Prevalence of Anxiety Symptoms and Its Associated Factors in Older Adults: A Systematic Review and Meta‐Analysis,” Journal of General and Family Medicine 26 (2024): 116–127. 40061394 10.1002/jgf2.750 PMC11890046

[7] A. P. Association , “Diagnostic and Statistical Manual of Mental Disorders: DSM‐5‐TR /American Psychiatric Association. First, MB,” Penn State University Libraries Catalog (2014). Psu.edu, [cited 2025 Nov 20].

[8] A. M. Johnson , A. Kenny , L. Ramjan , T. Raeburn , and A. George , “Oral Health Knowledge, Attitudes, and Practices of People Living With Mental Illness: A Mixed‐Methods Systematic Review,” BMC Public Health 24 (2024): 2263. 39164704 10.1186/s12889-024-19713-1 PMC11337876

[9] R. Borg‐Bartolo , A. Roccuzzo , P. Molinero‐Mourelle , M. Schimmel , K. Gambetta‐Tessini , and A. Chaurasia , “Global Prevalence of Edentulism and Dental Caries in Middle‐Aged and Elderly Persons: A Systematic Review and Meta‐Analysis,” Journal of Dentistry 127 (2022): 104335. 36265526 10.1016/j.jdent.2022.104335

[10] J. Choi , J. Price , S. Ryder , D. Siskind , M. Solmi , and S. Kisely , “Prevalence of Dental Disorders Among People With Mental Illness: An Umbrella Review,” Australian and New Zealand Journal of Psychiatry 56 (2022): 949–963. 34461748 10.1177/00048674211042239

[11] T. Afroz , J. Beyene , K. Zaheer , M. Alameddine , M. H. Nabi , and M. D. H. Hawlader , “Oral Health Care Challenges in Individuals With Severe Mental Illness: A Qualitative Meta‐Synthesis,” Frontiers in Oral Health 6 (2025): 1655450. 41019451 10.3389/froh.2025.1655450 PMC12464039

[12] E. B. Paraguassu and J. P. Lacerda , “Oral Health of the Elderly in Brazil: Systematic Review,” Brazalian Journal of Implantology and Health Science 1 (2019): 25–33.

[13] B. C. Demográfico . Principais Resultados | IBGE [Internet] wwwibgegovbr. https://www.ibge.gov.br/estatisticas/sociais/populacao/9662‐censo‐demografico‐2010.html.

[14] Brazil IDHM UF 2010 | United Nations Development Programme [Internet]. UNDP. https://www.undp.org/pt/brazil/idhm‐uf‐2010.

[15] E. Von Elm , D. G. Altman , M. Egger , S. J. Pocock , P. C. Gøtzsche , and J. P. Vandenbroucke , “The Strengthening the Reporting of Observational Studies in Epidemiology (STROBE) Statement: Guidelines for Reporting Observational Studies,” Lancet 370 (2007): 1453–1457. 18064739 10.1016/S0140-6736(07)61602-X

[16] J. Colaço , F. Muniz , D. Peron , et al., “Oral Health‐Related Quality of Life and Associated Factors in the Elderly: A Population‐Based Cross‐Sectional Study,” Ciência & Saúde Coletiva 25 (2020): 3901–3912. 32997022 10.1590/1413-812320202510.02202019

[17] L. M. Stoffel , F. Muniz , P. R. Colussi , C. K. Rösing , and E. L. Colussi , “Nutritional Assessment and Associated Factors in the Elderly: A Population‐Based Cross‐Sectional Study,” Nutrition 55 (2018): 104–110. 29980089 10.1016/j.nut.2018.03.053

[18] Brazil MINISTÉRIO DA SAÚDE Manual do Instrumento de Avaliação da Atenção Primária à Saúde Primary Care Assessment Tool PCATool‐Brasil Brasília ‐DF. (2010). [Internet]. https://bvsms.saude.gov.br/bvs/publicacoes/manual_avaliacao_pcatool_brasil.pdf.

[19] O. Pereira D'Avila , E. Harzheim , L. Hauser , L. F. Pinto , E. D. D. Castilhos , and F. N. Hugo , “Validation of the Brazilian Version of Primary Care Assessment Tool (PCAT) for Oral Health‐PCATool Brazil Oral Health for Professionals,” Ciência & Saúde Coletiva 26 (2021): 2097–2108. 34231722 10.1590/1413-81232021266.23432020

[20] A. G. Lopes , X. Ju , L. Jamieson , and F. L. Mialhe , “Oral Health‐Related Quality of Life Among Brazilian Adults With Mental Disorders,” European Journal of Oral Sciences 129 (2021): e12774. 33786899 10.1111/eos.12774

[21] A. Bhatia , R. K. Sharma , S. Tewari , H. Khurana , and S. C. Narula , “Effect of Fluoxetine on Periodontal Status in Patients With Depression: A Cross‐Sectional Observational Study,” Journal of Periodontology 86 (2015): 927–935. 25812910 10.1902/jop.2015.140706

[22] D. Trindade , R. Carvalho , V. Machado , L. Chambrone , J. J. Mendes , and J. Botelho , “Prevalence of Periodontitis in Dentate People Between 2011 and 2020: A Systematic Review and Meta‐Analysis of Epidemiological Studies,” Journal of Clinical Periodontology 50 (2023): 604–626. 36631982 10.1111/jcpe.13769

[23] C. X. W. Teoh , M. Thng , S. Lau , et al., “Dry Mouth Effects From Drugs Used for Depression, Anxiety, Schizophrenia and Bipolar Mood Disorder in Adults: Systematic Review,” BJPsych Open 9 (2023): e53. 36938801 10.1192/bjo.2023.15 PMC10044002

[24] I. Cannon , A. Robinson‐Barella , G. McLellan , and S. E. Ramsay , “From Drugs to Dry Mouth: A Systematic Review Exploring Oral and Psychological Health Conditions Associated With Dry Mouth in Older Adults With Polypharmacy,” Drugs & Aging 40 (2023): 307–316. 36943673 10.1007/s40266-023-01017-5

[25] S. E. Niklander , M. Martínez , A. Miranda , and M. Rodriguez , “Treatment Alternatives for Dry Mouth: A Scoping Review,” Journal of Clinical and Experimental Dentistry 14 (2022): e846. 36320676 10.4317/jced.59912 PMC9617261

[26] Z. Zhu , X. Qi , Y. Zheng , Y. Pei , and B. Wu , “Age Differences in the Effects of Multi‐Component Periodontal Treatments on Oral and Metabolic Health Among People With Diabetes Mellitus: A Meta‐Epidemiological Study,” Journal of Dentistry 135 (2023): 104594. 37355088 10.1016/j.jdent.2023.104594 PMC10437212

[27] L. P. Weijdijk , L. Ziukaite , G. A. Van der Weijden , E. W. Bakker , and D. E. Slot , “The Risk of Tooth Loss in Patients With Diabetes: A Systematic Review and Meta‐Analysis,” International Journal of Dental Hygiene 20 (2022): 145–166. 33973353 10.1111/idh.12512 PMC9291053

[28] M. S. Kirkman , V. J. Briscoe , N. Clark , et al., “Diabetes in Older Adults,” Diabetes Care 35 (2012): 2650–2654. 23100048 10.2337/dc12-1801 PMC3507610

[29] E. S. M. Dos Santos , R. de Oliveira Máximo , F. B. de Andrade , C. de Oliveira , M. F. Lima‐Costa , and T. da Silva Alexandre , “Differences in the Prevalence of Prediabetes, Undiagnosed Diabetes and Diagnosed Diabetes and Associated Factors in Cohorts of Brazilian and English Older Adults,” Public Health Nutrition 24 (2021): 4187–4194. 32972476 10.1017/S1368980020003201 PMC9014254

[30] F. Remelli , M. G. Ceresini , C. Trevisan , M. Noale , and S. Volpato , “Prevalence and Impact of Polypharmacy in Older Patients With Type 2 Diabetes,” Aging Clinical and Experimental Research 34 (2022): 1969–1983. 35723858 10.1007/s40520-022-02165-1 PMC9464133

[31] S. M. Marcus , K. B. Kerber , A. J. Rush , et al., “Sex Differences in Depression Symptoms in Treatment‐Seeking Adults: Confirmatory Analyses From the Sequenced Treatment Alternatives to Relieve Depression Study,” Comprehensive Psychiatry 49 (2008): 238–246. 18396182 10.1016/j.comppsych.2007.06.012 PMC2759282

[32] F. Mehrabi , P. Amiri , P. Naseri , and F. Azizi , “Factors Associated With Depression, Anxiety, and Stress in Men and Women: Findings From a Population‐Based Study in Iran,” Archives of Iranian Medicine 25 (2022): 533–541. 37543875 10.34172/aim.2022.85 PMC13661072

[33] A. E. Thompson , Y. Anisimowicz , B. Miedema , W. Hogg , W. P. Wodchis , and K. Aubrey‐Bassler , “The Influence of Gender and Other Patient Characteristics on Health Care‐Seeking Behaviour: A QUALICOPC Study,” BMC Family Practice 17 (2016): 38. 27036116 10.1186/s12875-016-0440-0 PMC4815064

[34] S. Blom , F. Lindh , A. Lundin , B. Burström , G. Hensing , and J. Löve , “How Gender and Low Mental Health Literacy Are Related to Unmet Need for Mental Healthcare: A Cross‐Sectional Population‐Based Study in Sweden,” Archives of Public Health 82 (2024): 12. 38273389 10.1186/s13690-023-01228-7 PMC10809616

